# Spatial and spatiotemporal patterns of human visceral leishmaniasis in an endemic southeastern area in countryside Brazil

**DOI:** 10.1590/0037-8682-0702-2021

**Published:** 2022-04-29

**Authors:** Cleya da Silva Santana Cruz, Diogo Tavares Cardoso, Claudio Luiz Ferreira, David Soeiro Barbosa, Mariângela Carneiro

**Affiliations:** 1 Universidade Federal de Minas Gerais, Faculdade de Medicina, Programa de Pós-Graduação em Infectologia e Medicina Tropical, Belo Horizonte, MG, Brasil.; 2 Secretaria de Estado da Saúde de Minas Gerais, Diamantina, MG, Brasil.; 3 Universidade Federal de Minas Gerais, Instituto de Ciências Biológicas, Programa de Pós-Graduação em Parasitologia, Belo Horizonte, MG, Brasil.; 4 Universidade Federal de Ouro Preto, Núcleo de Pesquisas em Ciências Biológicas, Programa de Pós-Graduação e Doenças Parasitárias, Ouro Preto, MG, Brasil.

**Keywords:** Visceral leishmaniasis, Spatial analysis, Spatio-temporal analysis, Epidemic, Endemic diseases

## Abstract

Visceral leishmaniasis (VL) has shown endemic pattern and epidemic episodes in urban and rural areas, however, there are still gaps in knowledge with regards to disease transmission. This study aimed to analyze the spatiotemporal dispersion of VL cases in the municipality of Araçuaí, Minas Gerais. A study of confirmed VL cases was conducted considering the endemic and epidemic periods between 2012 and 2017. The incidence rate was calculated, and for spatial analysis, the kernel map, directional distribution ellipse, and space-time scanning techniques were used. The correlations between VL cases and exposure variables (precipitation, humidity, and temperature) were calculated. The mean incidence of VL in the endemic period was 18.5 (95% confidence interval (CI) 5.9-32.5) and 44.4 in the epidemic period (95%CI, 12.0-28.6) by 100,000 inhabitants. The relative risk for the epidemic period was 2.4 (95% CI 1.4-4.1) when compared to the endemic period. A higher incidence of the disease was observed in rural areas of the municipality. Kernel mapping analysis revealed hotspots in the urban area of the municipality. The directional distribution ellipse encompasses the urban perimeter and part of the rural area of the municipality, expanding eastward during the epidemic period. Spatial analysis revealed a high-risk cluster for VL in rural areas. A positive correlation was observed between VL cases and temperature during the endemic period. Spatial analysis allowed us to outline the epidemiological scenario of human VL cases. These findings may be useful in case surveillance and in the work of health professionals and managers in Brazil.

## INTRODUCTION

Visceral leishmaniasis (VL) is a neglected tropical disease with worldwide prevalence of which it is endemic in 70 countries on five continents^1^ ^,^ ^2^. In the Americas, VL is caused by *Leishmania infantum*, which belongs to the *Leishmania donovani* complex and is transmitted mainly by *Lutzomyia longipalpis*. Domestic dogs are considered to be the main parasitic reservoirs in urban environments and play an important role in the transmission cycle ^3^.

The urbanization of VL in Brazil intensified in the 1980s, coinciding with the first urban epidemic recorded in Teresina, the capital of Piauí State^4^. The factors associated with the urbanization process, its characterization, and implications for the control of VL still represent challenges^5^. The processes of urbanization, propagation, and dissemination of the disease may be related to multiple and complex conditions, among which are the migration process, with disorderly occupation, precarious living and housing conditions^6^, the adaptation of the vector to peridomiciliary conditions^7^, as well as the control of vectors and reservoirs, and also the availability of susceptible or infected canine reservoirs^8^. These processes reflect the annual incidence rates in Brazil, ranged from 1.7 to 2.6 per 100,000 inhabitants between 1990 and 2016^9^.

In Brazil, control strategies are based on the Visceral Leishmaniasis Surveillance and Control Program guidelines, with measures aimed at reducing morbidity and case-fatality rates through early diagnosis and treatment of human cases, decreasing the risk of transmission, controlling the population of domestic reservoirs and vectors, and promoting health education^10^. However, even with the implementation of the program, an expansion of VL cases in some endemic areas^11^ and the emergence of epidemic processes have been identified in Brazil^12^ ^-^ ^16^. The Visceral Leishmaniasis Surveillance and Control Program presents difficulties in the execution and development of its actions^3^ ^,^ ^11^ ^,^ ^17^, which is reflected in the expansion of the disease throughout the country. 

Despite the maintenance of endemic patterns and frequent epidemic episodes in different contexts, there are still gaps in knowledge regarding the characteristics of disease transmission in the urban environment that impact disease control. In addition, VL studies in epidemic situations have mostly been conducted in large municipalities. Therefore, studies are needed to investigate the expansion of the disease during endemic and epidemic periods in small cities so that disease control actions can be planned in these municipalities. This study aimed to perform a spatial and spatiotemporal analysis of endemic and epidemic patterns in the occurrence of human visceral leishmaniasis in Araçuaí, Minas Gerais, a small municipality in southeastern Brazil. 

## METHODS

### Design and study area

This spatial and spatiotemporal study focuses on the disease patterns of VL cases notified to the Notifiable Diseases Information System (SINAN) from the municipality of Araçuaí, Minas Gerais, between 2012 and 2017. 

The municipality of Araçuaí is located northeast of the state of Minas Gerais in the Jequitinhonha mesoregion. According to the last census conducted in 2010, its population is 36,013 inhabitants, distributed over a territorial unit of 2,236.28 km² ^18^. According to data from the Brazilian Institute of Geography and Statistics (IBGE) census (2010), 65.07% of the population in the municipality of Araçuaí live in urban areas, 38.3% of households have adequate sanitation, 53.6% of urban households are on tree-lined public streets, and only 5.3% of households are on adequately urbanized streets (presence of gutters, storm drains, sidewalks, pavements, and curbs).

### Source of data

This study considered confirmed VL cases reported to involving residents in the municipality of Araçuaí from 2012 to 2017. The cases were separated into two periods: 2012 to 2014, characterized as an endemic period, and 2015 to 2017, characterized as an epidemic period. Municipality and population data were obtained from the IBGE^18^. 

For the purpose of this study, an epidemic was defined as the occurrence of a health-related event exceeding normal expectations^19^ with the number of cases beyond what is expected in a specific area and time, in the presence of an epidemiological link^20^.

VL cases that occurred during the above-mentioned periods were georeferenced to obtain the exact locations, which were the residential addresses of the people with the disease, as recorded in the investigation form. A Garmin eTrex 30x Global Positioning System (GPS) device was used. The units of analysis were urban areas and 27 rural census sectors. The rural area consisted of localities and two districts: Engenheiro Schnoor and Itira. 

### Incidence rates and spatial analyses

The analyses contemplated that 1^st^ three-year period 2012-2014, and 2^nd^ three-year period 2015-2017. Incidence rates were calculated as follows: average incidence for each period, considering the population for each year. The estimates were based on the 2010 National Population Census conducted by the IBGE^18^. The calculation of incidence rates was used to promote greater stability in these rates, as this is a small-population municipality. The OpenEpi platform (Open Source Epidemiologic Statistics for Public Health), version 3.01, was used to calculate incidence rates and relative risks.

Kernel mapping has been used to identify areas with higher VL cases^21^. Each observation was adjusted based on the distance from the central value of the kernel. The basic parameter of this estimator is the radius of influence, which defines the neighborhood of the point to be interpolated and controls the “smoothing” of the generated surface, an estimation function with phenomenon-smoothing properties. This tool performs exploratory interpolation that generates a dense surface presenting hot spots where a high concentration of the disease occurs. For the density assessment, a radius of influence of 500 m was stipulated. 

A directional distribution ellipse was used to identify the expansion and orientation of clusters in the epidemic and endemic periods, marking the regions where the highest concentration of VL cases occurred. The major axis of the ellipse defines the direction of the maximum dispersion of the distribution, whereas the minor axis is perpendicular to the anterior axis and defines the minimum dispersion^22^. 

Space-time scanning techniques were used to detect risk clusters for VL. The significance test of the identified clusters was based on the comparison of a null distribution, which was obtained using Monte Carlo simulation. The relative risk of each cluster was calculated, which represents the ratio of the risk of the disease occurring within the cluster to those outside it^23^.

For the identification of space-time clusters, the following criteria were used: no geographical overlap of clusters, maximum cluster size equal to 50.0% of the exposed population, considering a maximum temporal size of 50.0% of the study period and time precision expressed in years, and circular clusters, significance level of 0.05, and 999 replications^24^. The significance test of the identified clusters was based on a comparison of a null distribution obtained using Monte Carlo simulation. Thus, different areas could be compared, and the software presented a relative risk^23^.

For the construction of the kernel maps and for the application of the directional distribution, QGIS® software version 2.18 was used, and SaTScan software, version 9.4.4, was used for the identification of spatial and spatiotemporal clusters. 

In addition, to elucidate possible explanatory variables in the incidence of VL in humans, the correlations between the environmental variables, precipitation, humidity, temperature, and the number of VL cases in the period were calculated. Environmental variables were selected based on the Meteorological Database for Teaching and Research of the National Institute of Meteorology (BDMEP/INMET). GraphPad Prism software (San Diego, USA, version 6.00) was used for the statistical analysis of the data. For the environmental variables, the Shapiro-Wilk normality test was used. Correlation analyses were performed using Spearman’s correlation test when the data did not show a normal distribution, whereas Pearson’s correlation test was used for normal distribution. The significance level was set at p < 0.05.

### Ethical considerations

This study was approved by the Research Ethics Committee of the Federal University of Minas Gerais (CAAE number 94476318.8.0000.5149). All data were analyzed with respect to patient anonymity.

## RESULTS

Between January 2012 and December 2017, 68 new cases of VL were reported in residents of the Araçuaí/Minas Gerais municipality. Of these, 20 cases occurred during the endemic period (2012-2014) and 48 cases occurred during the epidemic period (2015-2017). The number of confirmed VL cases and the annual incidence rates in the municipality of Araçuaí/Minas Gerais from 2012 to 2017 are shown in Table 1.

**TABLE 1:** Distribution of confirmed cases and incidence rates of visceral leishmaniasis from 2012 to 2017 in the municipality of Araçuaí, Minas Gerais, Brazil.

	Endemic period			Epidemic period		
Year	2012	2013	2014	2015	2016	2017
Number of confirmed cases	05	07	08	14	18	16
Incidence rates*	13.9	19.4	22.2	38.9	50.0	44.4
(95%CI)	(5.9-32.5)	(9.4-40.1)	(11.3-43.8)	(23.2-65.3)	(31.6-79.0)	(27.4-72.2)

*Incidence Rates per 100,00 inhabitants. **Source:** Notifiable Diseases Information System (SINAN - Municipal/ 2018).

The mean incidence of visceral leishmaniasis in the endemic period was 18.5 (95% confidence interval (CI) 5.9-32.5) and 44.4 in the epidemic period (95%CI 12.0-28.6) by 100,000 inhabitants. The relative risk for the epidemic period was 2.4 (95%CI 1.4-4.1) when compared to the endemic period. 

**FIGURE 1:**
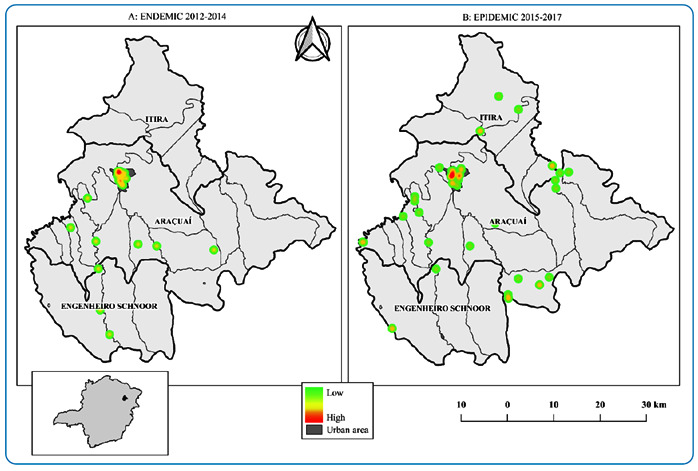
Kernel density map for cases of visceral leishmaniasis for the endemic (2012-2014) and epidemic (2015-2017) periods in the municipality of Araçuaí, Minas Gerais, Brazil. **Data source:** National Disease Notification System - SINAN.

The kernel map identified the regions where the highest concentration of cases occurred in the municipality. Hot spots (red) indicate a higher concentration of cases, whereas cold spots (green) indicate a lower concentration. The main hotspots in the two three-year periods were located in the urban area of the municipality (Figure 1). During the endemic period (2012-2014) there was a concentration of cases in the northern region of the municipality’s urban area. During the epidemic period (2015-2017), VL continued within the urban area, with a tendency to expand into the central region, as indicated by the hotspots (Figure 1). 

**FIGURE 2:**
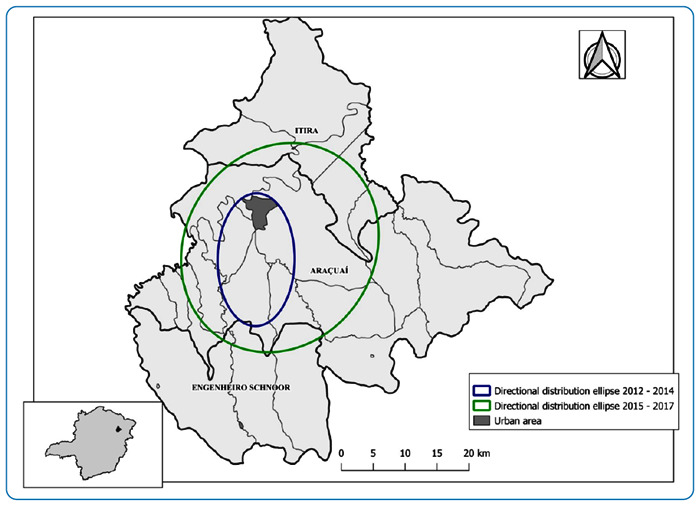
Directional distribution ellipse of visceral leishmaniasis cases for the endemic (2012-2014) and epidemic (2015-2017) periods in the municipality of Araçuaí, Minas Gerais, Brazil. **Data source:** National Disease Notification System - SINAN.

The directional distribution ellipse identified the expansion of VL cases in the Araçuaí municipality (Figure 2). During the endemic period (2011-2014), the ellipse encompassed the urban perimeter and a part of the rural area, returning to the southern part of the municipality. During the epidemic period (2015-2017), the ellipse increased, trending towards the east. A change can be observed in the mean center point of the ellipse from the endemic to the epidemic period. An increase in ellipse size during the epidemic period reinforced the dispersion of VL cases in the municipality. 

**FIGURE 3:**
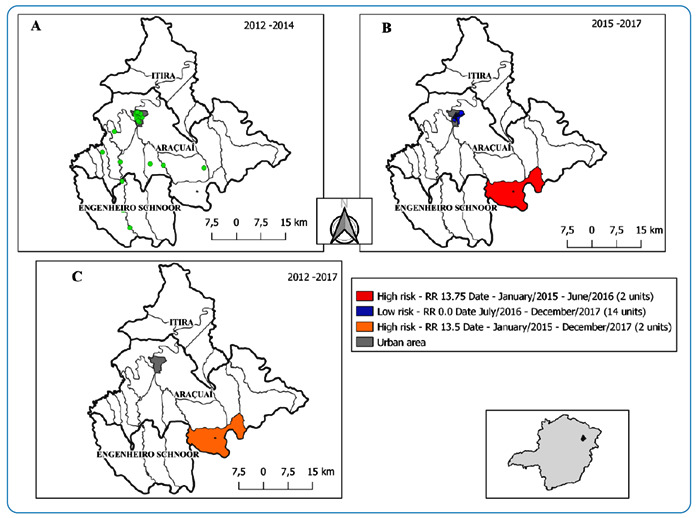
Scanning analysis and spatiotemporal clustering: **(A)** endemic period (2012-2014), **(B)** epidemic period (2015-2017), and **(C)** entire study period (2012-2017) for visceral leishmaniasis in the municipality of Araçuaí, Minas Gerais, Brazil. **Data source:** National Disease Notification System - SINAN.

The space-time scan in the endemic period did not identify high-risk clusters (Figure 3A). Two clusters were identified during the epidemic period (2015-2017). The figure shows a high-risk cluster between January 2015 and June 2016; therefore, people living in it are at a higher risk of acquiring VL than people living outside this area. This cluster was located in the southern part of the rural area of the municipality. A low-risk cluster in the urban area was detected during the endemic period (July/2016 - December 2017) (Figure 3B). The analysis detected a high-risk spatiotemporal cluster for VL in the southern (rural) part of the Araçuaí municipality. The cluster had a higher relative risk of acquiring VL than those living outside this area between January 2015 and December 2017 (Figure 3C).

Through the analysis of the endemic and epidemic periods, it was possible to observe that during the endemic period, the temperature correlated positively with the VL cases and the variables analyzed (Table 2). 

**TABLE 2:** Correlation between visceral leishmaniasis cases and humidity, precipitation and temperature according to endemic period (2012-2014) and epidemic period (2015-2017), in the municipality of Araçuaí, Minas Gerais, Brazil.

	Endemic period		Epidemic period	
Variables	r	p-value	r	p-value
Precipitation+	-0.1475	0.1954	0.1516	0.1887
Temperature*	0.3250	0.0266	0.2533	0.0775
Relative humidity of the air*	-0.1415	0.2051	-0.0430	0.4017

*Pearson’s and Spearman’s correlations.

## DISCUSSION

This study demonstrated a difference in the geographic distribution of VL in the municipality of Araçuaí during the endemic and epidemic periods. New foci of the disease occurred during the epidemic period, while the disease persisted in areas where it had occurred during the endemic period. This demonstrates that the current control measures may not be sufficient to control VL in municipalities. 

The ineffectiveness of the VL control strategy may be related to the lack of sustainability of a permanent surveillance system with extensive use of human and financial resources^25^. Studies have shown that most VL cases occur in urban areas^26^. However, in the results for the municipality of Araçuaí, VL was distributed in urban and rural areas during both the endemic and epidemic periods, with the disease spreading and shifting during the epidemic period.

Both the prevalence and incidence of VL depend on understanding the different forms of the disease associated with geographically isolated transmission cycles and regional differences in surveillance^27^. With regards to the expansion, some studies also indicate that the disease may be associated with the low impact of the control measures employed, possible improvement of the diagnosis and notification system, and people’s mobility^3^ ^,^ ^28^.

When demonstrating the evolution of the spatial distribution of VL in the urban area of the municipality of Araçuaí^29^ between 2007 and 2013, the spatial distribution of human and canine VL cases exhibited a significant aggregate pattern with the occurrence of human and canine infection in the central region of the city (urban area). In the present study, scanning analysis resulted in a low-risk cluster in the urban areas. 

If endemic and epidemic processes are not contained, there will likely be a tendency for VL to concentrate in both urban and rural areas of the municipality. The present study results corroborate the occurrence of outbreaks in urban areas in other municipalities, such as Teresina, Piauí^6^, and Araguaína, in Tocantins^30^, with a higher risk in rural areas. 

The directional distribution with the formation of an ellipse in the urban area shows that a higher population density may favor disease transmission in these areas. The ellipse does not cover the districts of the municipality of Araçuaí, but it includes rural communities that can be considered priority regions for surveillance and control of VL in the municipality. 

The higher relative risk of people from rural areas of Araçuaí acquiring VL compared with people living outside this area during the epidemic period is in opposition to the idea defended by some authors^28^ ^,^ ^31^. In these studies in medium and large municipalities, the paradigm of the typically rural disease was overcome, since in these cities, the zoonotic cycle of VL is established in urban and peri-urban areas. 

In Fortaleza, Ceará^15^ Fortaleza and Araguaína, Tocantins^32^, the average temperature had a negative influence on the incidence rate of VL. However, our study showed a positive correlation between temperature and VL.

VL transmission and vector proliferation are affected by multiple factors that determine the risk of contracting the disease, including temperature^33^. Temperature influences the sandfly population, which is an important factor in VL transmission. A study conducted in Tocantins showed that there seems to be an ideal temperature that can favor the occurrence of VL cases^32^. The temperatures related to VL were neither too high nor too low. 

One limitation of this study was the use of secondary data from SINAN. Although VL is a compulsory notification disease in Brazil, the possibility of under-reporting of cases cannot be ruled out. However, the extent of this underreporting is likely to be minimal since it covers all areas of the healthcare system (public and private) at various levels of complexity. Furthermore, it is important to emphasize that medication for therapeutic use is dispensed exclusively by the Brazilian Unified Health System (SUS) through a notification form, which should minimize the underreporting of cases. Another limitation of this study was the use of data from canine leishmaniasis. It is also noteworthy that the lack of access to databases of information regarding the results of examinations of canine surveys made it impossible to study the dog population and its influence on VL cases. Despite the high VL incidence rates in epidemic periods, the restricted number of cases limited the use of more statistically robust clustering techniques for spatial analysis in smaller areas.

Despite these limitations, with the use of spatial analysis techniques, this study was able to map areas of higher risk for VL in Araçuaí, the geographical expansion of the disease, and the occurrence and incidence of cases. In this way, spatial analysis techniques allow the identification of priority areas for control and surveillance, thus reducing the costs of the Visceral Leishmaniasis Surveillance and Control Program. Cost reduction may justify the application of spatial analysis to the control of VL^17^ ^,^ ^34^. 

Spatial analysis allowed us to outline the epidemiological scenario of human cases of VL in the municipality of Araçuaí during the endemic and epidemic periods. The number of VL cases in Araçuaí remains high, considering its incidence in Brazil. It is distributed in the urban and rural areas of the municipality, with expansion during the epidemic period. These results suggest that ideal conditions for establishing and maintaining transmission are found in these locations. In addition, the pattern of occurrence of VL is not static, and the disease may expand to other areas of the municipality. These findings may be useful in case surveillance and in the work of health professionals and managers as well as in guiding further research. 

## References

[1] Alvar J, Vélez ID, Bern C, Herrero M, Desjeux P, Cano J (2012). Leishmaniasis worldwide and global estimates of its
incidence. PLoS One.

[2] World Health Organization (WHO) (2021). Leishmaniasis.

[3] Romero GA, Boelaert M (2010). Control of visceral leishmaniasis in Latin América - a systematic
review. PLoS Negl Trop Dis.

[4] Costa CHN, Pereira HF, Araújo MV (1990). Epidemia de leishmaniose visceral no Estado do Piauí, Brasil,
1980-1986. Rev Saúde Pública.

[5] Werneck GL (2008). Forum: geographic spread and urbanization of visceral
leishmaniasis in Brazil. Introduction. Cad Saúde Pública.

[6] Almeida AS, Medronho R, Werneck G (2011). Identification of risk areas for visceral leishmaniasis in
Teresina, Piauí State, Brazil. Am J Trop Med Hyg.

[7] Rangel EF, Vilela ML (2008). Lutzomyia longipalpis (Diptera, Psychodidae, Phlebotominae) and
urbanization of visceral leishmaniasis in Brazil. Cad Saúde Pública.

[8] Nascimento EL, Martins DR, Monteiro GR, Barbosa JD, Ximenes MF, Maciel BL (2008). Forum: geographic spread and urbanization of visceral
leishmaniasis in Brazil. Postscript: new challenges in the epidemiology of
Leishmania chagasi infection. Cad Saúde Pública.

[9] Bezerra JMT, Araújo VEM, Barbosa DS, Martins-Melo FR, Werneck GL, Carneiro M (2018). Burden of leishmaniasis in Brazil and federated units, 1990-2016:
Findings from Global Burden of Disease Study 2016. PLoS Negl Trop Dis.

[10] Ministério da Saúde (MS). Secretaria de Vigilância em Saúde.
Sistenma Nacional de Vigilância em Saúde (2006). Manual de vigilância e controle da leishmaniose visceral.

[11] Zuben APB, Donalisio MR (2016). Dificuldades na execução das diretrizes do Programa de Vigilância
e Controle da Leishmaniose Visceral em grandes municípios
brasileiros. Cad. Saúde Pública.

[12] Cruz CSS, Barbosa DS, Oliveira VC, Cardoso DT, Guimarães NS, Carneiro M (2021). Factors associated with human visceral leishmaniasis cases during
urban epidemics in Brazil: a systematic review. Parasitology.

[13] Lima ALM, Lima ID, Coutinho JFV, Sousa UPST, Rodrigues MAG (2017). Changing epidemiology of visceral leishmaniasis in northeastern
Brazil: a 25-year follow-up of an urban outbreak. Trans R Soc Trop Med Hyg.

[14] Carranza-Tamayo CO, Werneck GL, Romero GAS (2016). Are opossums a relevant factor associated with asymptomatic
Leishmania infection in the outskirts of the largest Brazilian
cities?. Braz J Infect Dis.

[15] Freitas JCC, Sampaio-Filho AP, Santos GJLS, Lima AL, Nunes-Pinheiro DCS (2013). Analysis of seasonality, tendencies and correlations in human and
canine visceral leishmaniasis. Acta Sci Vet.

[16] Brazuna JCM, Silva EA, Brazuna JM, Domingos IH, Chaves N, Honer MR (2012). Profile and geographic distribution of reported cases of visceral
leishmaniasis in Campo Grande, State of Mato Grosso do Sul, Brazil, from
2002 to 2009. Rev Soc Bras Med Trop.

[1] Maia-Elkhoury ANS, Alves WA, Sousa-Gomes ML, Sena JM, Luna EA (2008). Visceral leishmaniasis in Brazil: trends and
challenges. Cad Saúde Pública.

[18] Instituto Brasileiro Geografia e Estatística (IBGE) (2010). Censo Cidades.

[19] Braga J, Werneck GL, Medronho RA, Vergetti KB, Raggio LR, Werneck GL (2008). Epidemiologia.

[20] Kane AJ, Morley PS (1999). Proceedings of the Annual Convention of the AAEP: How to Investigate a
disease outubreak: in 45^th^ AAEP Annual Convention.

[21] Ministério da Saúde (MS). Secretaria de Vigilância em Saúde (2007). Introdução à Estatística Espacial para a Saúde Pública.

[22] Yuill RS (1971). The standard deviational ellipse: an updated tool for spatial
description. Geogr Ann Ser B.

[23] Pellegrini A, Kulldorff M (2016). SaTScan - Manual do Usuário.

[24] Kulldorff M (1997). A spatial scan statistic. Commun Stat - Theory Methods.

[25] Werneck GL, Pereira TJCF, Farias GC, Silva FO, Chaves FC, Gouvêa MV (2008). Avaliação da efetividade das estratégias de controle da
leishmaniose visceral na cidade de Teresina, Estado do Piauí, Brasil:
resultados do inquérito inicial. Epidemiol Serv Saúde.

[26] Harhay MO, Olliaro PL, Costa DL, Costa CH (2011). Urban parasitology: visceral leishmaniasis in
Brazil. Trends Parasitol.

[27] Ready PD (2014). Epidemiology of visceral leishmaniasis. Clinical Epidemiol.

[28] Furtado AS, Nunes FBBF, Santos AM, Caldas AJM (2015). Análise espaço-temporal da leishmaniose visceral no estado do
Maranhão, Brasil. Ciênc Saúde Coletiva.

[29] Ursine RL, Dias JVL, Morais HA, Pires HHR (2016). Human and canine visceral leishmaniasis in an emerging focus in
Araçuaí, Minas Gerais: spatial distribution and socio-environmental
factors. Mem Inst Oswaldo Cruz.

[30] de Toledo CRS, de Almeida AS, Chaves SAM, Sabroza PC, Toledo LM, Caldas JP (2017). Vulnerability to the transmission of human visceral leishmaniasis
in a Brazilian urban area. Rev Saúde Pública.

[31] Dantas-Torres F, Brandão-Filho SP (2006). Expansão geográfica da leishmaniose visceral no Estado de
Pernambuco. Rev Soc Brasil Med Trop.

[32] Oliveira IBB, Batista HL, Peluzio JM, Pfrimer IAH, Rodrigues FM, Carmo-Filho JR (2014). Epidemiological and environmental aspects of visceral
leishmaniasis in children under 15 years of age between 2007 and 2012 in the
City of Araguaína, State of Tocantins, Brazil. Rev Soc Bras Med Trop.

[33] Monteiro EM, Silva JCF, Costa RT, Costa DC, Barata RA, Paula EV (2005). Leishmaniose visceral: estudo de flebotomíneos e infecção canina
em Montes Claros, Minas Gerais. Rev Soc Bras Med Trop.

[34] Otranto D, Dantas-Torres F (2013). The prevention of canine leishmaniasis and its impact on public
health. Trends Parasitol.

